# Strain-dependent disease progression and necrotizing granuloma formation in a murine model induced by virulent strains of *Mycobacterium avium* complex

**DOI:** 10.1128/spectrum.03128-25

**Published:** 2026-03-24

**Authors:** Haruka Hikichi, Shiho Omori, Hajime Nakamura, Shintaro Seto, Koji Furuuchi, Kozo Morimoto, Minako Hijikata, Naoto Keicho

**Affiliations:** 1 Department of Pathophysiology and Host Defense, The Research Institute of Tuberculosis, Japan Anti-Tuberculosis Association 46635 , Tokyo, Japan; 2 Department of Basic Mycobacteriosis, Nagasaki University Graduate School of Biomedical Sciences 200674 , Nagasaki, Japan; 3 Respiratory Disease Center, Fukujuji Hospital, Japan Anti-Tuberculosis Association 469425, Tokyo, Japan; 4 Department of Clinical Mycobacteriosis, Nagasaki University Graduate School of Biomedical Sciences 200674 , Nagasaki, Japan; 5 The Research Institute of Tuberculosis, Japan Anti-Tuberculosis Association 46635 , Tokyo, Japan; Rutgers New Jersey Medical School, Newark, New Jersey, USA

**Keywords:** *Mycobacterium avium *complex, mouse model, granuloma, necrotizing granuloma, drug susceptibility

## Abstract

*Mycobacterium avium* complex (MAC) is the leading cause of non-tuberculous mycobacterial pulmonary disease (NTM-PD), a chronic infection with a heterogeneous clinical course. Although murine models of MAC-PD exist, faithfully reproducing the progressive pathology and variable treatment responses of the disease remains challenging. Here, we assessed the virulence of five clinical MAC strains in immunocompetent BALB/c mice, including a newly identified highly virulent isolate, NBRC112750. Two strains, FKJ-1 and NBRC112750, induced progressive pulmonary infection characterized by rising bacterial loads and extensive lung involvement over 25 weeks postinfection. In BALB/c mice, both strains produced necrotizing granulomas resembling those observed in *M. tuberculosis*-infected C3HeB/FeJ mice, characterized by neutrophilic infiltration, foamy macrophages, and collagen encapsulation. We further established an inhalation-based infection model using FKJ-1, in which low-dose exposure reproducibly generated necrotizing granulomas. Despite demonstrating *in vitro* drug susceptibility, FKJ-1 responded poorly to standard antimicrobial, therapy indicating strain-dependent variability in treatment efficacy. Together, these findings establish a murine model that accurately reflects the critical pathological and therapeutic features of MAC-PD and provides a valuable platform for studying MAC pathogenesis and evaluating novel therapies.

**IMPORTANCE:**

The global incidence of pulmonary disease (PD) caused by non-tuberculous mycobacteria, particularly Mycobacterium avium complex (MAC), is increasing. However, the mechanisms underlying its pathological heterogeneity and variable treatment outcomes remain poorly understood. Here, we establish a murine model that recapitulates the key features of progressive MAC-PD, including necrotizing granuloma formation. We also demonstrate strain-specific differences in treatment responses despite comparable *in vitro* drug susceptibility. Notably, highly virulent strains induced necrotizing granulomatous lesions similar to those observed in patients with tuberculosis or MAC-PD. This study provides a valuable *in vivo* platform for investigating host-pathogen interactions, elucidating strain-dependent pathogenesis, and optimizing treatment strategies for MAC-PD.

## INTRODUCTION

Pulmonary infection caused by non-tuberculous mycobacteria (NTM), referred to as NTM pulmonary disease (NTM-PD), is a chronic respiratory condition that has gained increasing prevalence worldwide (1–3). Among NTM species, *Mycobacterium avium* complex (MAC), comprising *M. avium* and *M. intracellulare*, is the leading cause of NTM-PD in several countries, including Japan (2, 4–6).

NTM-PD, including MAC-PD, presents a heterogeneous clinical spectrum, with two primary radiological phenotypes recognized: the fibrocavitary (FC) and the nodular bronchiectatic (NB) types, each associated with distinct demographics and disease courses (4, 7). The FC type, characterized by the occurrence of cavitary lesions predominantly in the upper lobes, is more prevalent among men with underlying or prior PDs, including pulmonary tuberculosis and chronic obstructive PD. In contrast, the NB type typically affects post-menopausal women without a history of smoking. The FC type is associated with poorer treatment response and worse prognosis, whereas the NB type has a higher risk of post-treatment NTM-PD recurrence (8). Despite these variations, both share a common pathological feature: granulomatous inflammation throughout the airways (9, 10), which is implicated in MAC-PD pathogenesis. Such peribronchial granulomatous inflammation may damage the airway walls, cause bronchial distortion and eventually lead to irreversible airway structural changes such as bronchiectasis.

The introduction of macrolide-based combination therapy, typically including clarithromycin (or azithromycin), ethambutol, and rifampicin, has markedly improved treatment success compared to earlier regimens (4, 7, 8). However, recurrence and persistence of infection remain common, and treatment outcomes vary among patients, suggesting that host–pathogen interactions play a critical role in disease progression and therapeutic responses (11, 12).

Several murine models of pulmonary MAC infection have been established to better understand the pathogenesis of MAC-PD and develop more effective treatments (12, 13). Immunocompromised mouse strains, such as *Nos2* knockout, nude, or beige, can sustain chronic MAC infection. However, the absence of adaptive immunity limits the relevance of findings in them to humans. Infection with specific clinical MAC isolates can be established in immunocompetent mice, but it typically results in non-necrotizing granulomatous inflammation, which may not fully replicate the pathological heterogeneity observed in patients.

Given the chronically progressive nature of MAC-PD, it can be hypothesized that long-term infection in a murine model could better explain disease progression and pathological heterogeneity in patients, including the development of necrotizing granulomas (9, 14, 15). In our previous study, clinical MAC strains, FKJ-1, FKJ-2, FKJ-5, and FKJ-8, were identified as exhibiting persistent or highly proliferative infection in mouse lungs (16). In the present study, we extended this approach by incorporating long-term observation up to 25 weeks and by adding a newly identified highly virulent isolate, NBRC112750, to further evaluate strain-dependent pathogenic diversity. Furthermore, we established an aerosol infection model that could reproduce a broader range of pathological features, with which we further evaluated the *in vivo* responses to therapy modeled on the standard regimen for MAC-PD. This approach identified MAC strains capable of inducing chronic infection and necrotizing granulomas. The results obtained demonstrate strain-dependent variability in therapeutic outcomes, thereby providing a murine model that more faithfully reflects the MAC-PD pathological spectrum.

## RESULTS

### Virulence of the *M*. *intracellulare* strain NBRC112750 in mice

**Fig 1:**
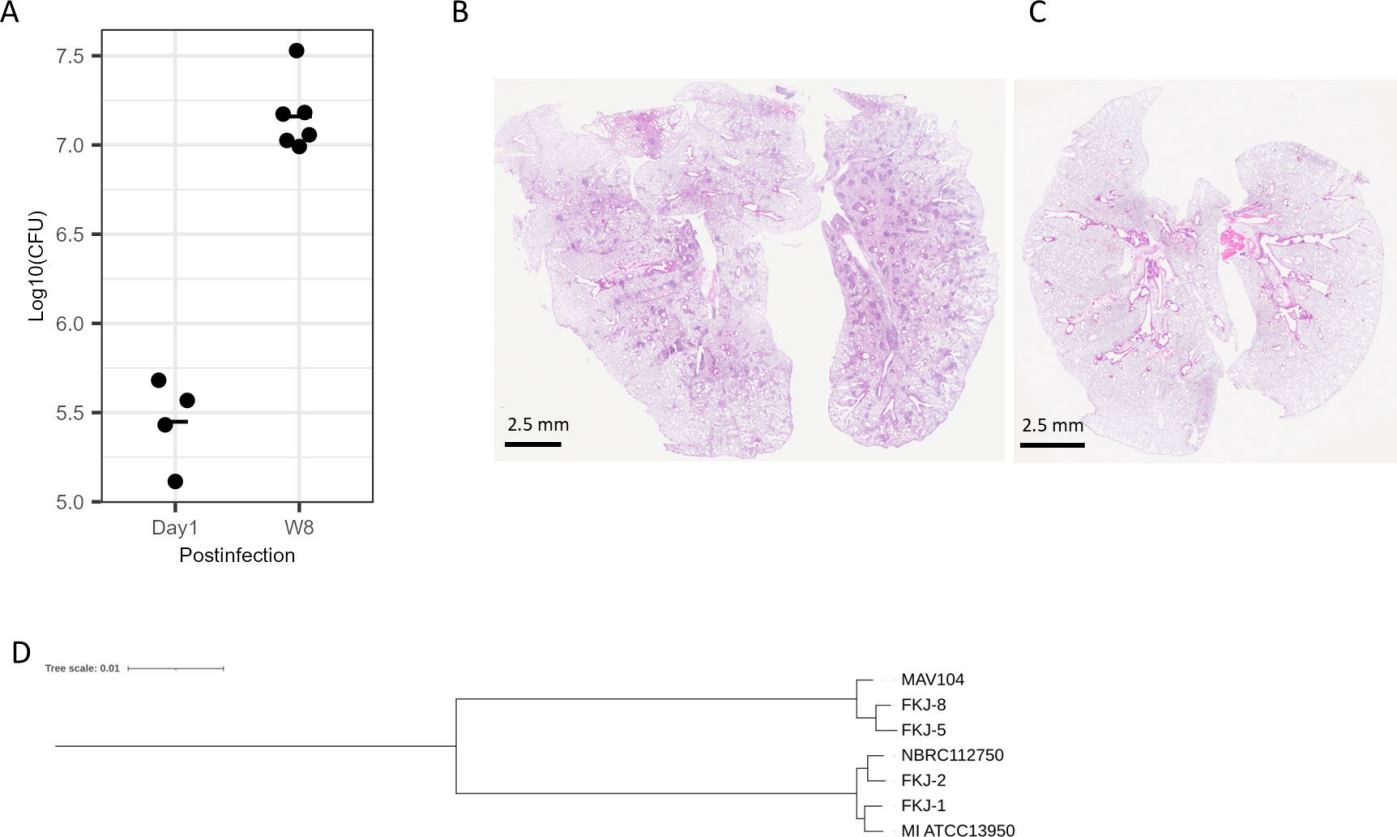
Mouse model infected with *M. intracellulare* strain NBRC112750. (**A**) Mycobacterial burden in BALB/c mouse lungs. CFU count at each time point, and their means are shown (one day, *n* = 4; 8 weeks, *n* = 6). Statistical comparisons were performed using the Mann–Whitney *U* test, *P* = 0.0105. (**B and C**) A representative image of the H&E-stained whole lung lobes isolated from NBRC112750-infected (**B**) and non-infected (**C**) BALB/c mice at 8 weeks p.i. (**D**) A phylogenetic tree of the clinical *M. avium* complex (MAC) strains used in this study. A phylogenetic tree was constructed based on the whole-genome sequences of the five strains studied, along with two reference MAC strains. MAV104, *M. avium* 104; MI ATCC13950, *M. intracellulare* ATCC 139500.

We collected clinical MAC strains from three public microbial repositories in Japan: the Japan Collection of Microorganisms, the Gene Bank project of the National Agriculture and Food Research Organization, and the Biological Resource Center of the National Institute of Technology and Evaluation. Strains for evaluation in a mouse infection model were selected based on the following criteria: (i) isolation from patients with MAC-PD, including sources such as sputum or bronchoalveolar lavage fluid; (ii) the ability to induce pulmonary granulomatous lesions following intranasal infection in BALB/c mice; and (iii) sustained or increasing mycobacterial burdens in the lungs at 8 weeks postinfection (p.i.). We screened the MAC strains as previously described (16) and confirmed the virulence of selected ones in BALB/c mice. Among the 18 strains tested, only NBRC112750 met all three criteria. At 8 weeks p.i. with NBRC112750, BALB/c mice exhibited peribronchial granulomas with elevated mycobacterial burdens in the lungs (Fig. 1A and B), whereas non-infected mice showed no granulomatous lesions throughout the entire lung lobes and no detectable bacterial burden (Fig. 1C). Whole-genome sequencing confirmed that NBRC112750 was a strain of *M. intracellulare*. Whole-genome sequence-based phylogenetic analyses further demonstrated that NBRC112750 and four clinical isolates, FKJ-1, FKJ-2, FKJ-5, and FKJ-8, were genetically distinct, supporting their uniqueness within the MAC strains (Fig. 1D).

### PD progression in MAC-infected mouse models

**Fig 2:**
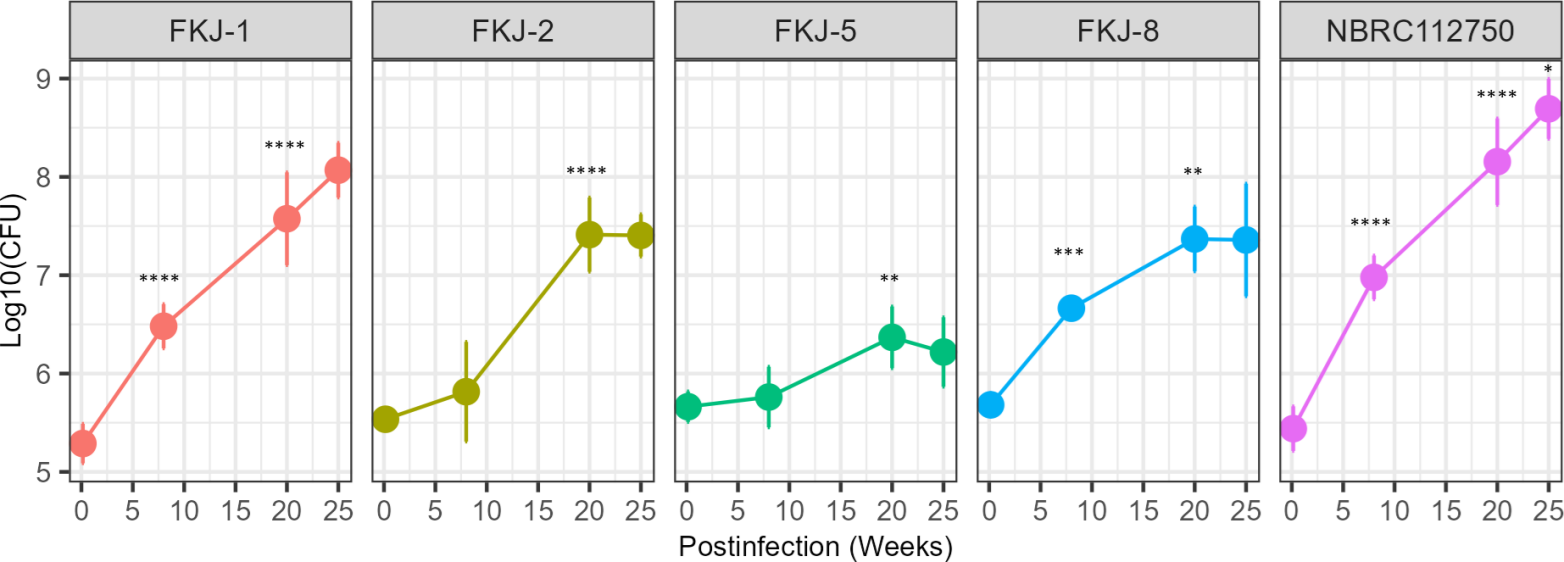
Mycobacterial burden of infected lungs over a 25-week infection period. BALB/c mice were intranasally infected with 10^6^ CFU of specific MAC strains and monitored for 25 weeks. At each time point, the mean ± SD of the CFU counts from six mice is shown. Multiple comparisons were conducted using the Tukey–Kramer post hoc test. The adjusted *P* values (adjp) for the comparisons between one time point and the immediate predecessor are indicated on the graphs (****adjp <10^−4^; ***adjp <10^−3^; **adjp <0.01; *adjp <0.05). Additional statistical comparisons made are presented in Table S1.

**Fig 3:**
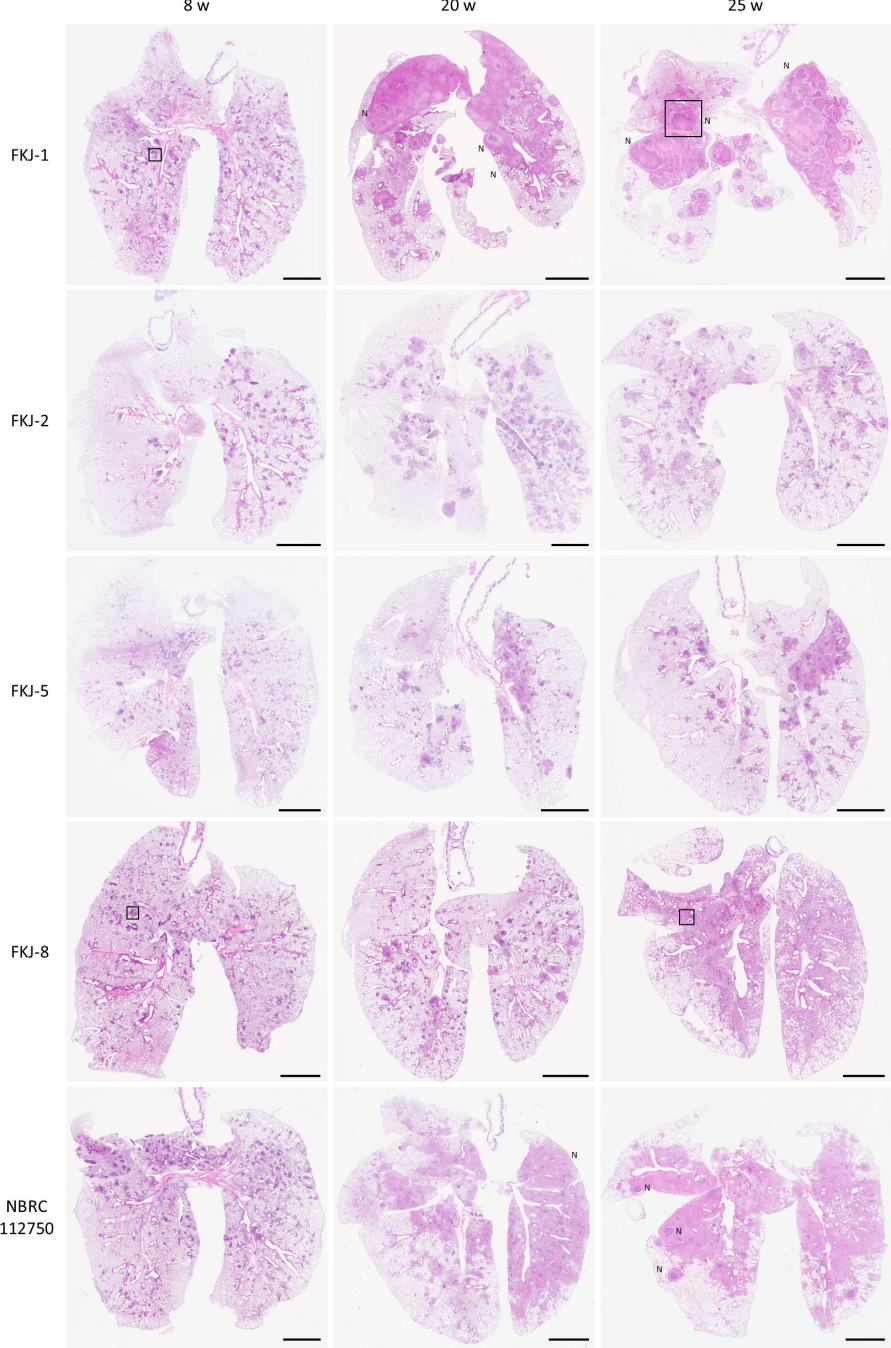
Development of mycobacterial granulomatous lesions in mouse lungs. Representative hematoxylin and eosin (H&E)-stained images of whole lung lobes isolated from BALB/c mice infected with the indicated MAC strains at respective time points (*n* = 3–4). Areas within boxes are shown at higher magnification in Fig. 4. N, necrotizing granuloma. Scale bar, 2.5 mm.

We previously isolated four clinical MAC strains that, upon intranasal administration into BALB/c mice, caused granulomatous lesions and increased or sustained the mycobacterial burden of the lungs (16). In this study, we evaluated disease progression in mouse models infected with five MAC strains, namely, FKJ-1, FKJ-2, FKJ-5, FKJ-8, and NBRC112750, over 24 weeks. FKJ-1, FKJ-2, and NBRC112750 were identified as *M. intracellulare*, while FKJ-5 and FKJ-8 were recognized as *M. avium*. The MAC strains were intranasally administered to BALB/c mice at a dose of 10^6^ CFU. All strains induced a progressive increase in lung mycobacterial burden throughout the infection period. In particular, the pulmonary burden of FKJ-1 and NBRC112750 exceeded 10^8^ CFU by 25 weeks of infection (Fig. 2). Histological observations revealed that all strains induced the formation of peribronchial granulomatous lesions at 8 weeks p.i. (Fig. 3), as previously described (16). By 20 weeks p.i., the lungs of mice infected with FKJ-1 and NBRC112750 exhibited extensive inflammatory consolidation across whole lobes and necrotizing granulomas with central necrotic cores, which eventually developed into well-defined necrotized granulomas (Fig. 3; Fig. S1). Among the other strains, although FKJ-2 and FKJ-5 increased bacterial burden and granuloma formation, disease progression was limited. In contrast, the bacterial burden of FKJ-8 initially increased similar to that of FKJ-1 and NBRC112750. However, the overall bacterial load for FKJ-8 remained consistently lower than those of FKJ-1 and NBRC112750 at the later stage of infection. Furthermore, the pathological changes induced by FKJ-8 did not progress to necrotizing granulomas, even during long-term infection. Considering these variations in bacterial dynamics and pathological progression, subsequent experiments focused on a comparative analysis of the infectivity of FKJ-1 and FKJ-8 strains.

### Necrotizing granuloma formation in the lungs of FKJ-1-infected mice

**Fig 4:**
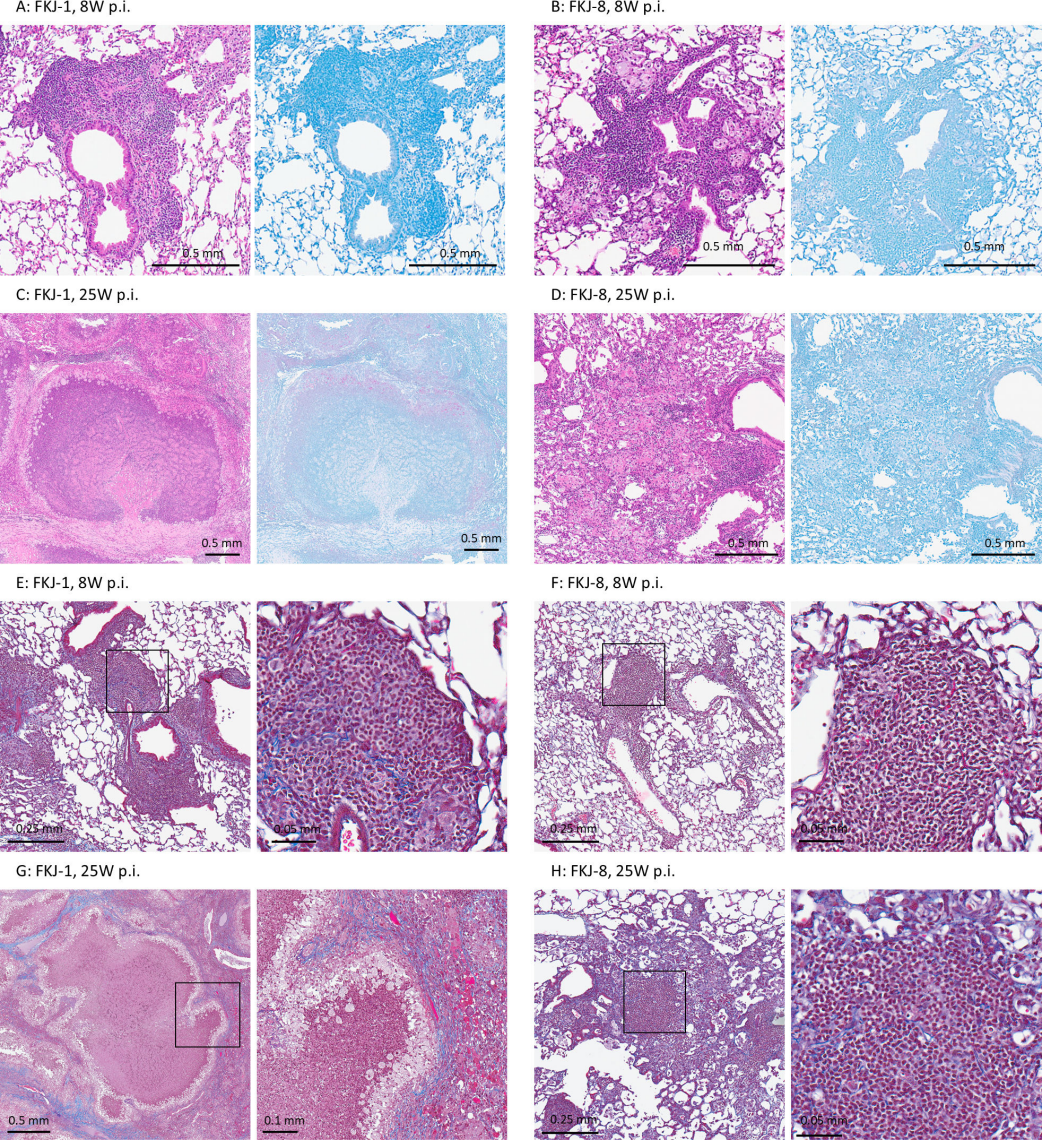
Histopathological features of granulomatous lesions in MAC-infected BALB/c mouse lungs. (**A–D**) H&E (Left) and Ziehl–Neelsen (ZN, right) staining of the lung granulomas in BALB/c mice infected with FKJ-1 at 8 weeks postinfection (p.i.) (**A**), FKJ-8 at 8 weeks p.i. (**B**), FKJ-1 at 25 weeks p.i. (**C**), and FKJ-8 at 25 weeks p.i. (**D**). (**E–H**) Masson’s trichrome staining of the granulomas indicates collagen distribution within the lungs of mice infected with FKJ-1 or FKJ-8 at 8 or 25 weeks p.i. Right panels show enlarged views of the boxed areas within the left panels.

We characterized the granulomas observed in the lungs of MAC-infected BALB/c mice (Fig. 4). Although both FKJ-1 and FKJ-8 induced peribronchial granulomas at 8 weeks p.i., FKJ-1-infected lungs exhibited necrotizing granulomas at 25 weeks p.i. (Fig. 4A through D). Ziehl–Neelsen staining identified mycobacterial presence within the epithelioid macrophages surrounding the necrotic cores. Masson’s trichrome staining further showed that FKJ-1-induced necrotizing granulomas were well-organized and encapsulated by collagen fibers at 25 weeks p.i. In contrast, granulomas induced by FKJ-1 infection at 8 weeks p.i. and by FKJ-8 infection at either time point exhibited collagen deposition without clear encapsulation (Fig. 4E through H). These results indicate that FKJ-8 infection did not induce necrotizing granulomas under the prevailing conditions, whereas FKJ-1 infection led to their formation at later stages in BALB/c mice.

**Fig 5:**
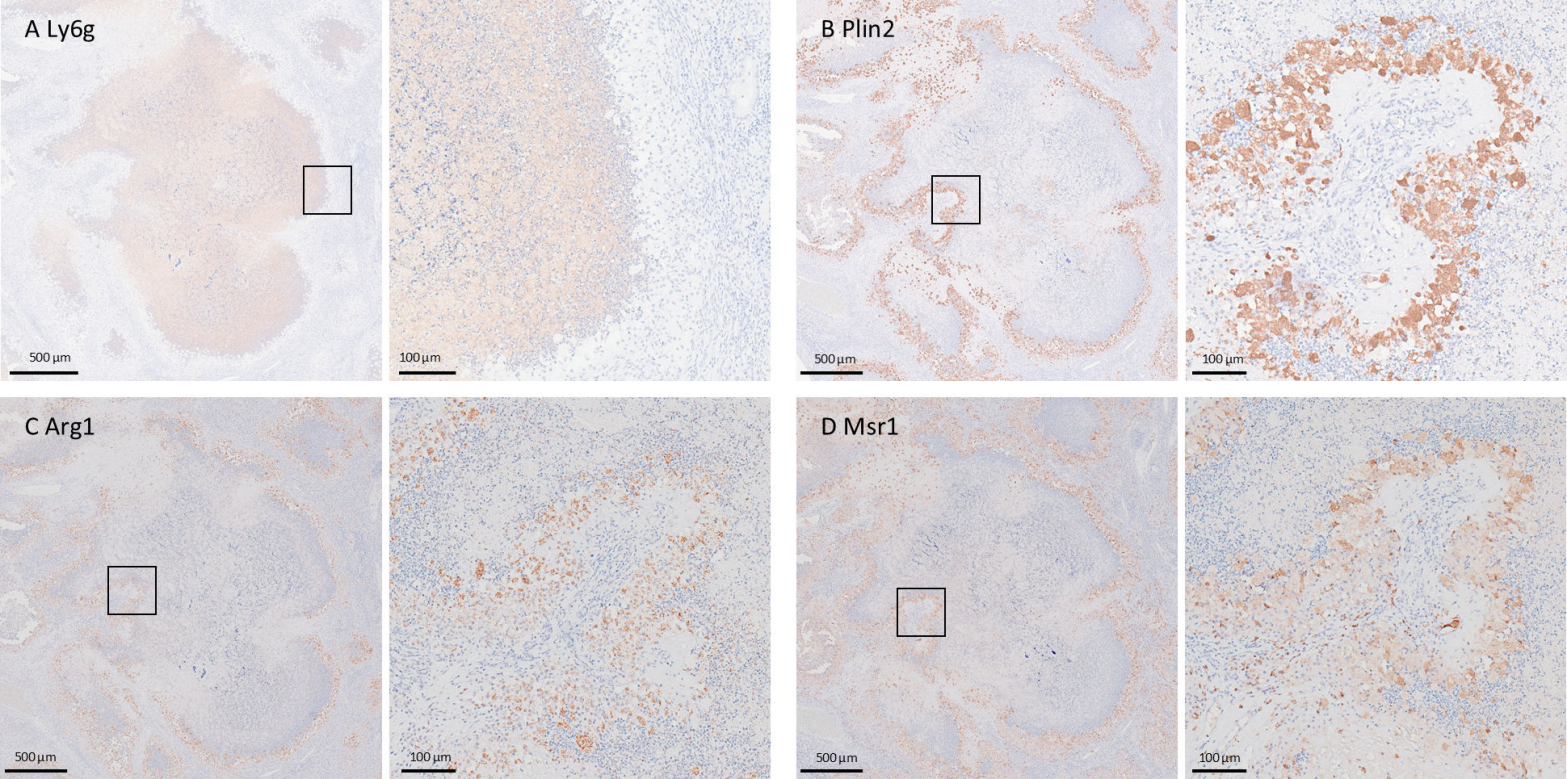
Localization of neutrophil- and foamy macrophage-specific markers within necrotizing granulomatous lesions in the lungs of BALB/c mice infected with FKJ-1. Immunohistochemistry (IHC) analysis of lesions from the lungs of BALB/c mice infected with the FKJ-1 strain at 25 weeks p.i., showing the neutrophil marker Ly6G (**A**) and foamy macrophage markers Plin2 (**B**), Arg1 (**C**), and Msr1 (**D**). Right panels display enlarged images of the corresponding boxed areas in the left panels.

To ascertain the cell composition of the FKJ-1-induced necrotizing granulomas in BALB/c mice, we examined neutrophil accumulation within them. Immunostaining for Ly6G, a neutrophil marker, revealed strong signals within the necrotic cores, indicating substantial neutrophil infiltration of necrotizing granulomas (Fig. 5A). Additionally, immunostaining signals for foamy macrophage markers Plin2, Msr1, and Arg1, which are foamy macrophage markers (17–19), localized to cells surrounding necrotic granulomas developed in FKJ-1-infected mice (Fig. 5B through D). Together, these findings suggest that chronic FKJ-1 infection in BALB/c mice leads to the development of necrotizing granulomas with a cell composition similar to that observed in *M. tuberculosis*-infected C3HeB/FeJ mice, as well as observed in tuberculosis and MAC-PD patients (9, 17, 20–26).

### Establishment of a novel mouse model via exposure to an aerosolized MAC strain

**Fig 6:**
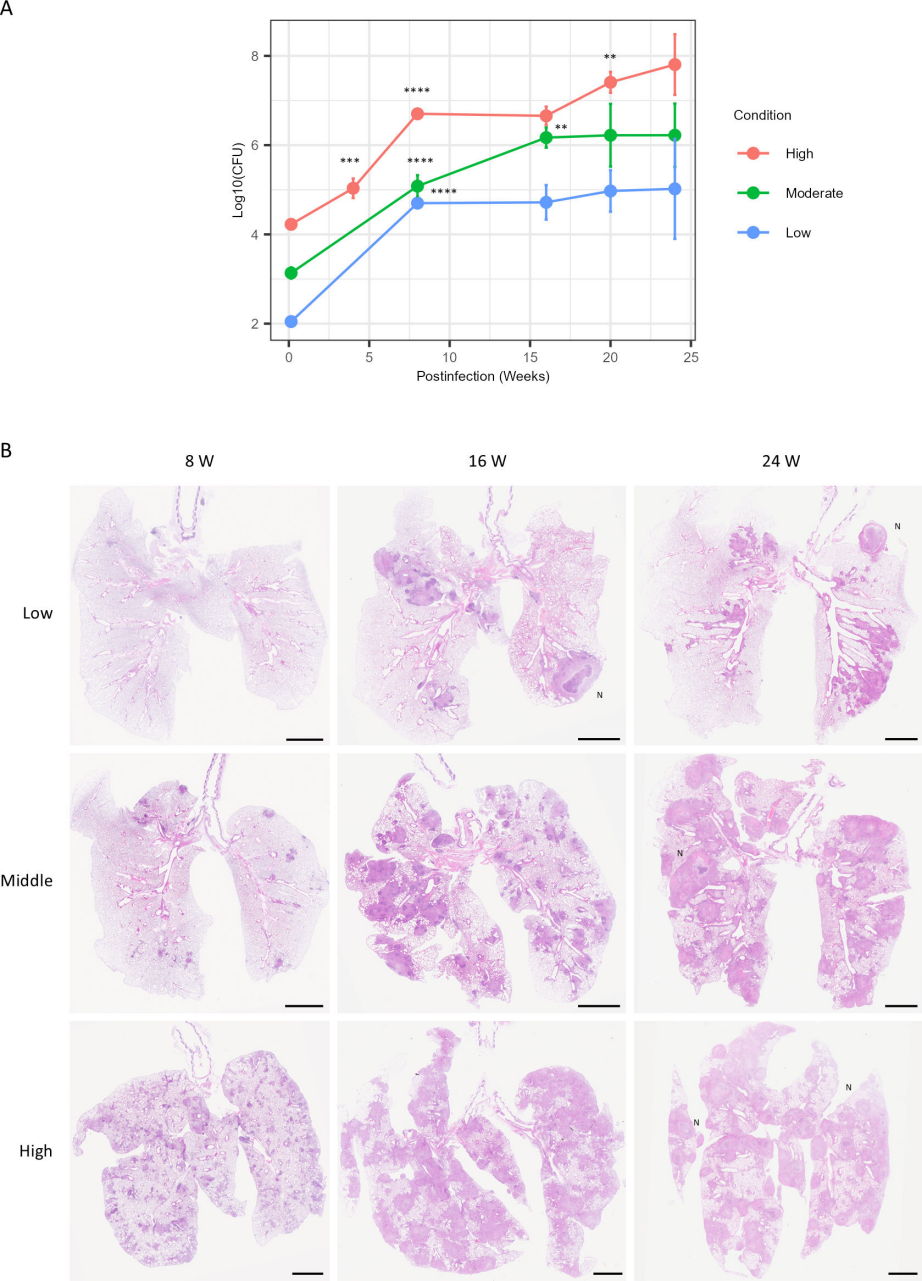
Aerosol infection of BALB/c mice with a specific MAC strain. BALB/c mice were infected with three doses of the FKJ-1 strain via aerosol exposure and monitored for 24 weeks. (**A**) Mycobacterial burden in mouse lungs. At each time point, the mean ± SD of CFU counts from six mice is shown. Mice were infected with 10^2^ (low), 10^3^ (moderate), and 10^4^ (high) CFU. Multiple comparisons were performed employing the Tukey–Kramer *post hoc* test. Adjp for comparisons between one time point and the immediate predecessor are indicated on the graphs (****adjp < 10^−4^, ***adjp < 10^−3^, and **adjp < 0.01. Results of additional statistical comparisons performed are presented in Table S1. (**B**) Development of mycobacterial granulomatous lesions in mouse lungs. Each panel shows a representative H&E-stained image of whole lung lobes from mice infected with the FKJ-1 strain at the indicated dose and time point. N, necrotizing granuloma. Scale bar, 2.5 mm.

We assessed the virulence of the FKJ-1 strain in BALB/c mice after administration via the aerosol route using an inhalation exposure system (Fig. 6). Mice were infected with doses of 10^2^ (low), 10^3^ (moderate), and 10^4^ (high) CFU, and the mycobacterial burdens as well as pathological features were monitored for up to 24 weeks. Lung mycobacterial burdens exhibited clear dose-dependent dynamics during the study period (Fig. 6A). Mice infected with low or moderate doses demonstrated increased bacterial burdens for up to 8 or 16 weeks p.i., respectively, and then plateaued. In contrast, high doses induced sustained elevations in lung mycobacterial burdens throughout the study, indicating progressively developing chronic infection.

Histopathologically, all lung lobes of high-dose-infected mice exhibited numerous lesions at 8 weeks p.i. In contrast, only a few lesions were found in low-dose-infected mice (Fig. 6B). By 16 or 24 weeks p.i., moderate- or high-dose-infected mice developed granulomatous lesions throughout whole lobes. At 24 weeks p.i., the necrotized cores appeared within these granulomatous lesions. Notably, distinct necrotizing granulomas were also observed in low-dose-infected mice at 16 weeks p.i. (Fig. 6B; Fig. S1).

### Therapeutic efficacy based on the standard MAC-PD regimen in MAC aerosol-infected mouse models

**Fig 7:**
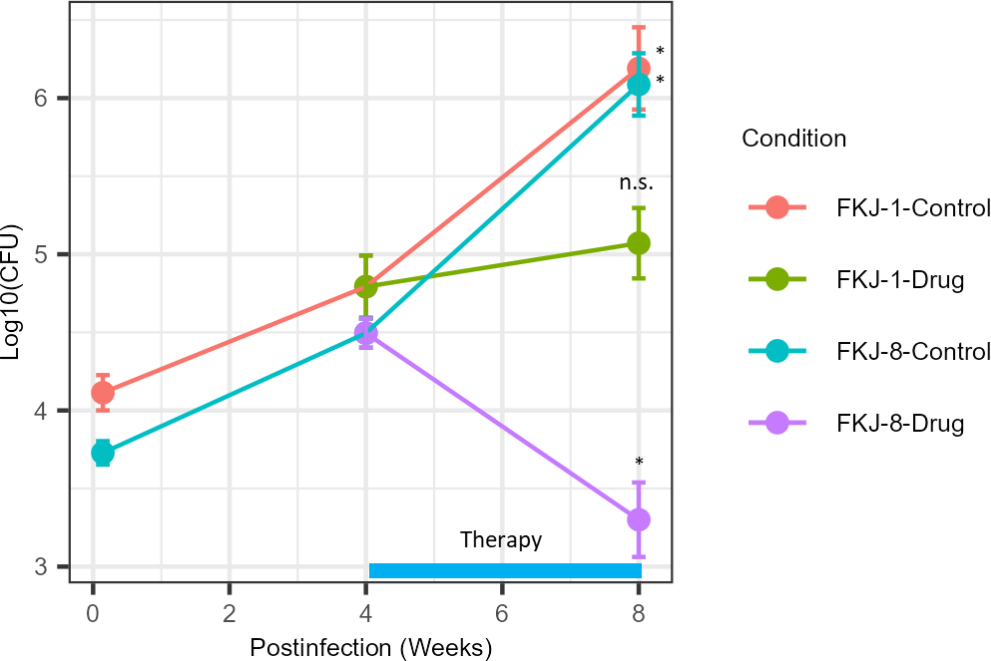
Treatment of MAC-infected mice. BALB/c mice were infected with the FKJ-1 or FKJ-8 strains via aerosol exposure. At 4 weeks p.i., the infected mice were treated with a combination of rifampicin (10 mg/kg/mouse), ethambutol (100 mg/kg/mouse), and clarithromycin (100 mg/kg/mouse), administered 5 days per week for 4 weeks. At each time point, the mean ± SD of the CFU counts from six mice is shown. The Tukey–Kramer *post hoc* test was used for multiple comparisons. Adjp at 4 and 8 weeks were compared and indicated on the graphs (*adjp < 0^−7^; n.s., not significant). Findings of additional statistical comparisons are presented in Table S1. The data are representative of two independent experiments.

We evaluated the therapeutic efficacy based on a standard MAC-PD regimen, comprising clarithromycin, ethambutol, and rifampicin, in mouse models infected with the MAC strains via exposure to the FKJ-1 and FKJ-8 aerosols (Fig. 7). The minimum inhibitory concentrations (MICs) of these antibiotics against the strains were determined and are listed in Table 1. BALB/c mice were infected with FKJ-1 or FKJ-8 at a high dose (10^4^ CFU). At 4 weeks p.i., chemotherapeutic treatment was introduced and continued for 4 weeks. The mycobacterial burdens in drug-treated FKJ-8-infected mice were significantly reduced at 8 weeks p.i., compared with both the baseline of treatment at 4 weeks p.i. (1.19-fold log_10_ reduction) and untreated controls at 8 weeks p.i. (2.39-fold log_10_ reduction). In contrast, FKJ-1-infected mice exhibited only a modest reduction at 8 weeks p.i. compared with untreated controls (1.03-fold log_10_ reduction). Bacterial burdens in the drug-treated mice at 8 weeks p.i. remained relatively stable compared with the treatment baseline at 4 weeks p.i. (0.34-fold log_10_ increase; adjusted *P* = 0.201). These findings suggest that FKJ-1 is less susceptible to the standard regimen *in vivo* despite demonstrating similar *in vitro* antibiotic sensitivity to FKJ-8.

**TABLE 1:** Minimum inhibitory concentrations of selected antibiotics against the FKJ-1 and FKJ-8 strains

Strain	MIC (μg/mL)		
	Clarithromycin	Ethambutol	Rifampicin
FKJ-1	0.5	2	0.25
FKJ-8	2	8	0.25

### Limited PD progression of FKJ-1 infection in C3HeB/FeJ mice

**Fig 8:**
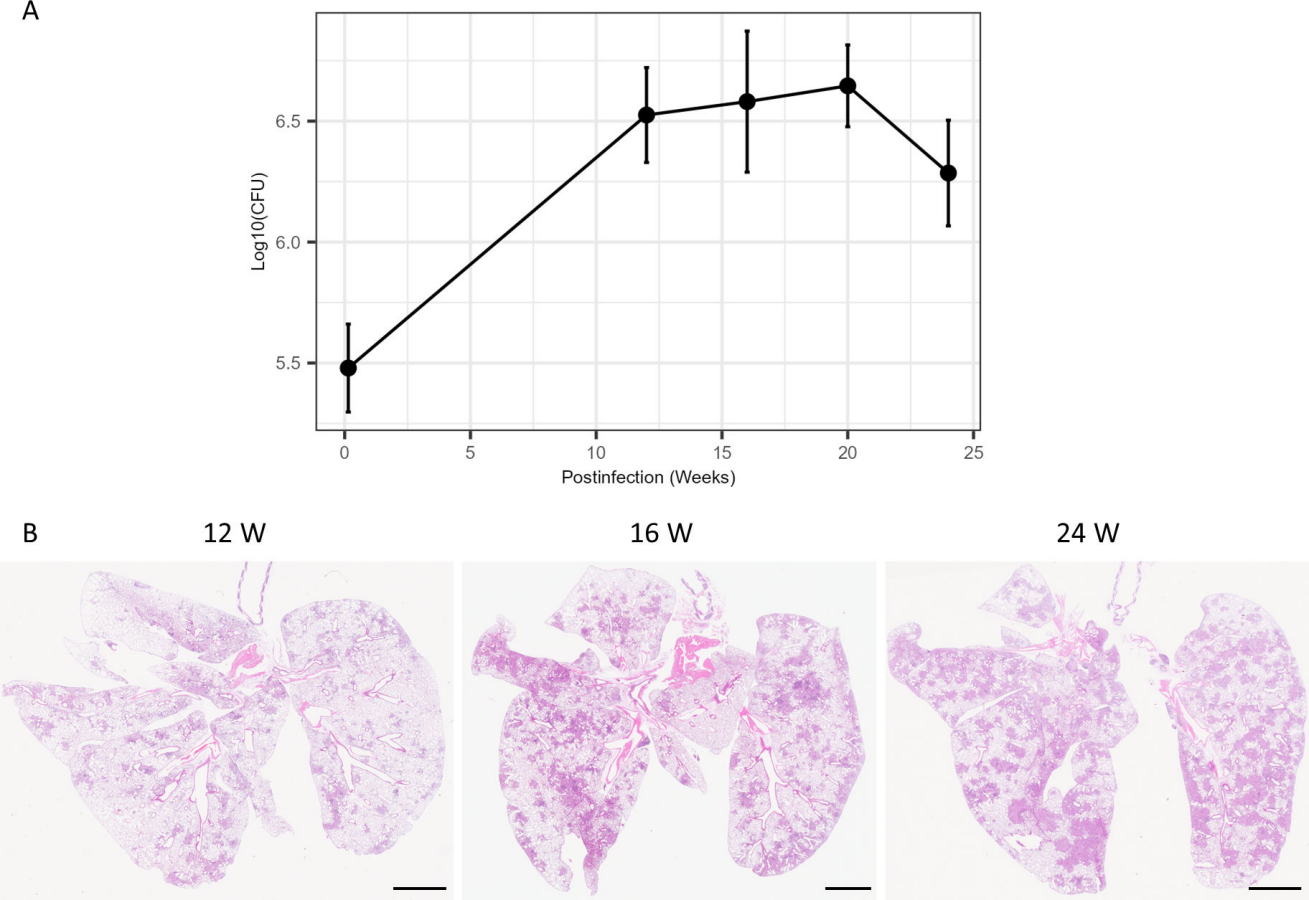
Infection of C3HeB/FeJ mice with FKJ-1. C3HeB/FeJ mice were infected with 10^6^ CFU of the FKJ-1 via the intranasal route and monitored for 24 weeks. (**A**) Mycobacterial burden in mouse lungs. At each time point, the mean ± SD of the CFU counts (*n* = 4 at day 1; *n* = 6 at other time points) is shown. Multiple comparisons were performed using the Tukey–Kramer *post hoc* test. Statistical comparison results are presented in Table S1. (**B**) The development of mycobacterial granulomatous lesions within mouse lungs. Each panel shows a representative H&E-stained image of whole lung lobes infected with the FKJ-1 strain at the indicated time point. Scale bar, 2.5 nm.

Infection of C3HeB/FeJ mice with *M. tuberculosis* induces the formation of necrotizing granulomas in the lungs (24, 25, 27). We evaluated long-term disease progression in C3HeB/FeJ infected with FKJ-1 (Fig. 8). C3HeB/FeJ mice were infected intranasally with 10^6^ CFU of FKJ-1 and monitored for 24 weeks. Pulmonary bacterial burden increased significantly at 12 weeks p.i. compared with the initial time point; however, this increase subsequently plateaued (Fig. 8A). At 24 weeks p.i. bacterial burden of FKJ-1 in C3HeB/FeJ mice (2.1 × 10^6^ CFU) was significantly lower than that observed in BALB/c mice (1.4 × 10^8^ CFU; Welch’s *t*-test, *P* < 10^−5^). C3HeB/FeJ mice infected with FKJ-1 exhibited peribronchial granulomas in the lungs at early time point similar to BALB/c mice infected with FKJ-1 and other MAC strains (Fig. 8B). However, these mice did not develop necrotizing granulomas at later stages. These results demonstrated that C3HeB/FeJ mice exhibit limited PD progression following FKJ-1 infection compared with BALB/c mice.

## DISCUSSION

This study identified a highly virulent MAC strain, NBRC112750, capable of inducing a chronic and progressive lung infection in BALB/c mice. To evaluate the pathological heterogeneity of MAC-PD, we infected immunocompetent mice with five MAC strains, including NBRC112750 and four previously isolated ones (16), and monitored their pathogenicity over a prolonged period. Necrotizing granulomas are widely regarded as precursors of cavitary lesions in tuberculosis and represent a transitional stage from caseous necrosis to cavity formation (28). Because cavitary lesions are associated with a more severe clinical phenotype and reduced treatment responsiveness in patients with MAC-PD (4, 8), the identification of MAC strains capable of inducing necrotizing granulomas in murine models is valuable for assessing advanced pathological states. Given the variable clinical course of MAC-PD, ranging from stable disease to progressive respiratory failure, we aimed to investigate whether such heterogeneity could be recapitulated in a mouse model.

A remarkable outcome of chronic infection with FKJ-1 or NBRC112750 was the development of necrotizing granulomas in the lungs (Fig. 3, Fig. S1). By 20-25 weeks p.i., BALB/c mice infected with either strain exhibited extensive inflammatory consolidation and necrotizing granulomas with central necrotic cores. In contrast, FKJ-2 and FKJ-8 induced non-necrotic granulomas despite lung mycobacterial burdens >10⁷ CFU. The necrotizing granulomas formed in FKJ-1 or NBRC112750-infected BALB/c mice resembled those observed in *M. tuberculosis*-infected C3HeB/FeJ mouse models (17, 23–25, 27).

In this study, we focused on FKJ-1 and FKJ-8 to further characterize the strain-dependent development of necrotizing granulomas in BALB/c mice. Among the strains capable of inducing necrotizing granulomas, FKJ-1 demonstrated the high reproducibility: all infected mice for pathological analysis (*n* = 4) consistently developed necrotizing granulomas by 25 weeks p.i., whereas one of the NBRC112750-infected mice (*n* = 4) failed to develop despite exhibiting high bacterial burden. Moreover, the necrotizing granulomas formed in FKJ-1–infected mice exhibited more distinct and organized pathological structures compared with those induced by NBRC112750 (Fig. 3). Conversely, FKJ-8 was selected as the representative non-necrotizing strain due to its bacterial proliferation kinetics during the first 8 weeks closely resembled those of FKJ-1 and NBRC112750, whereas FKJ-2 and FKJ-5 showed slower increases in bacterial burden. The early proliferation rate of FKJ-8 during the early infection period allowed us to compare later-stage pathological divergence between strains with comparable initial infectivity. Thus, FKJ-1 and FKJ-8 provided the most appropriate pair of strains for dissecting the strain-dependent mechanisms underlying necrotizing vs non-necrotizing granuloma formation in BALB/c mice.

We further characterized the necrotizing granulomas induced by FKJ-1 in BALB/c and observed that (i) neutrophil accumulation within the necrotic cores; (ii) expression of foamy macrophage markers, including Plin2, Msr1, and Arg1, in the epithelioid macrophages surrounding the necrotic areas. Although these markers are not exclusively macrophage-specific, their expression patterns identified foamy macrophages (17, 18); and (iii) encapsulation of the granulomas with collagen fibers (Fig. 3 and 4). These histopathological features were also observed in C3HeB/FeJ mice infected with *M. tuberculosis* (17, 18). In that model, necrotizing granulomas were attributed to reduced expression of *Sp110* and *Sp140*, which encode negative regulators of type I interferon expression (29–31). However, the development of similar necrotizing granulomas in BALB/c mice infected with MAC strains suggests the involvement of a distinct mechanism. Our findings suggest that in BALB/c mice, persistent local inflammation with abundant neutrophils and foamy macrophages may also contribute to the necrotizing pathology observed in tuberculosis mouse model.

We established a novel inhalation model of MAC infection via exposure to FKJ-1 aerosols using BALB/c mice (Fig. 6). Notably, low-dose FKJ-1 infection induced necrotizing granulomas at 16 weeks p.i., much earlier than moderate- or high-dose infections. This finding suggests that lesion development may depend on the dynamics of host–pathogen interactions rather than on the initial bacterial load. In the low-dose infection model, the bacteria gradually replicate, allowing prolonged immune activation, resulting in sustained inflammation and the formation of necrotizing granulomas. These observations suggest that chronic infection by certain virulent MAC strains can drive necrotizing granulomatous inflammation.

Moreover, the differential therapeutic responses induced by FKJ-1 and FKJ-8 highlight the importance of identifying strain-specific virulence determinants and treatment susceptibility. Several studies have demonstrated the efficacy of chemotherapeutic treatments in MAC-infected mouse models (32–35). However, despite similar *in vitro* antibiotic sensitivities, FKJ-1 infection resulted in persistent mycobacterial burdens *in vivo* during treatment (Fig. 7). Although lesion architecture did not show marked differences between FKJ-1 and FKJ-8 at earlier time points, subsequent granuloma organization or bacterial physiological states may influence drug penetration and tolerance. These possibilities are hypothetical and require further investigation. These findings suggest that our mouse model recapitulates the variable treatment responses observed in patients with MAC-PD, where certain clinical strains respond poorly to standard therapy despite confirmed *in vitro* susceptibility (8, 36). To clarify the design of the therapeutic evaluation in this study, we selected FKJ-1 and FKJ-8 not to compare species-level differences between *M. intracellulare* and *M. avium*, but to assess strain-dependent variability in treatment responses. Among the non-necrotizing granuloma-forming strains, FKJ-8 exhibited early bacterial proliferation in BALB/c mice that closely resembled FKJ-1 via intranasal infection, whereas FKJ-2 and FKJ-5 showed substantially slower increases (Fig. 2). FKJ-1 and FKJ-8 also displayed comparable proliferation kinetics in the aerosol infection model (Fig. 7). In addition, both strains demonstrated similar *in vitro* MIC profiles (Table 1), allowing us to evaluate *in vivo* drug responses without confounding differences in baseline drug susceptibility. These considerations made FKJ-1 and FKJ-8 the most suitable combination for investigating strain-dependent variation in antibiotic responsiveness in BALB/c mice.

To further investigate host susceptibility, we infected the C3HeB/FeJ mice with FKJ-1 (Fig. 8). The initial bacterial burden in these mice was similar to that in the BALB/c mice, but the pulmonary bacterial burden did not increase during the later infection period, and necrotizing granulomas failed to develop. These findings suggest that C3HeB/FeJ mice, despite their susceptibility to *M. tuberculosis*, could control FKJ-1 infection. One possible explanation is the involvement of *Nramp1* (*Slc11a1*), which encodes a phagosomal iron transporter known to modulate the survival of *M. avium* within macrophages (37). C3H-derived strains such as C3HeB/FeJ carry the functional allele, whereas BALB/c and C57BL/6 mice harbor a non-functional variant leading to enhanced mycobacterial susceptibility (38, 39). Our observations on FKJ-1 infection of C3HeB/FeJ mice raise the possibility that *Nramp1*-mediated restriction may reduce inflammation and lesion progression, thereby limiting the development of necrotic granulomas. Notably, aerosol infection of C3HeB/FeJ mice with a certain clinical MAC isolate was reported to induce necrotizing granulomatous lesions (40), reminiscent of the pathological features described in the same mouse model of *M. tuberculosis* infection (41). In contrast, a recent reproducibility study did not observe necrotizing granulomatous lesions following intratracheal inoculation with the same MAC isolate (42). Differences in infection routes may influence the kinetics of bacterial burden in the lungs, potentially leading to divergent pathological outcomes.

Overall, our findings highlight a complex interplay between bacterial virulence, host genetics, and persistent inflammatory responses that drives necrotizing granuloma development and treatment variability in MAC-PD. The murine model presented here replicates the key pathological and therapeutic aspects of the disease, representing a valuable platform for exploring host–pathogen interactions and optimizing more effective, strain-specific treatment strategies.

### Limitations

Several limitations of this study should be acknowledged. First, we did not perform a detailed analysis of polymorphisms or virulence-associated genes among the clinical isolates. Second, we did not characterize the cellular composition of granulomatous lesions, including necrotizing granulomas. Third, the mechanisms underlying strain-dependent differences in *in vivo* drug responses, including drug penetration and bacterial physiological state, remain speculative. Finally, limited information was available on the strain-dependent relationships between granulomatous lesion development and *in vivo* drug efficacy.

## MATERIALS AND METHODS

### Mice

BALB/c and C3HeB/FeJ mice were purchased from Japan SLC and Jackson Laboratory (https://www.jax.org/strain/000658), respectively. C3HeB/FeJ mice were maintained in a laminar air-flow cabinet and provided sterile bedding, water, and mouse chow in the animal facility at RIT. Specific pathogen-free status was verified by testing sentinel mice housed within the colony. Purchased BALB/c mice (6 weeks old) and in-house-maintained C3HeB/FeJ mice (6–10 weeks old) were transferred to the biosafety level (BSL) II or III animal facility of the RIT for intranasal or aerosol infections, respectively. In both facilities, mice were maintained in negative-pressure ventilated racks and provided with sterile bedding, water, and mouse chow during the experimental period.

### Infection with the MAC strains

The clinical MAC strains, FKJ-1, FKJ-2, FKJ-5, FKJ-8 (16), and NBRC112750, isolated from human tracheal lavage fluid, were cultured at 37°C in 7H9 medium supplemented with 10% Middlebrook ADC (BD Bioscience), 0.5% casamino acid, and 0.05% Tween 80 (mycobacterial medium) (31). NBRC112750 was provided by the National Institute of Technology and Evaluation. Single-cell suspensions were prepared as previously described (16) and stored at −80°C until use. For the infection experiments, the frozen stocks were thawed and kept on ice until inoculation without prior broth subculture. Mice were infected with the MAC strains either intranasally at a dose of 10^6^ CFU in 30 μL of saline or via the aerosol route using an infection exposure system (Glas-Col) at doses of 10^2^, 10^3^, or 10^4^ CFU to the lungs. All experiments were performed 2–3 times to ensure reproducibility. In uninfected control mice, no mycobacterial burden was detected at 25 weeks, and lung appearance remained unchanged between day 1 and week 25 (data not shown).

### Genome sequencing and phylogenetics

NBRC112750 genomic DNA and sequence libraries were prepared as previously described (16). Phylogenetic trees were constructed employing the Maximum-Likelihood method implemented in REALPHY software version 1.12 with default parameters (43). The GenBank genome sequences of *M. avium* 104 (accession number: CP000479) and *M. intracellulare* ATCC 13,950 (accession number: CP003322) were used as references. Whole-genome sequence data of the other MAC strains were accessed from the DRA database (accession number: DRA013277) (16).

### Pulmonary mycobacterial burden and histopathological analysis

At each pre-determined time point, the infected mice were euthanized by exsanguination under anesthesia using 0.75 mg/kg medetomidine, 4.0 mg/kg midazolam, and 5.0 mg/kg butorphanol administered via the intraperitoneal route. To determine pulmonary mycobacterial burden, lungs were homogenized using a FastPrep-24 5G instrument (MP Biomedicals). The homogenates were serially diluted and plated on 7H10 or 7H11 agar plates supplemented with 10% Middlebrook OADC (BD Bioscience) and 0.5% glycerol. For histological analysis, the whole lung lobes from the infected mice were fixed with 10% formalin in PBS for >24 h at room temperature. Tissue sections were stained with hematoxylin and eosin (H&E), Ziehl–Neelsen (ZN), or Masson’s trichrome staining for collagen detection. Immunohistochemistry was performed as previously described (9, 17, 18). Briefly, whole lung sections were deparaffinized and subjected to antigen retrieval in citrate or Tris buffer. After blocking with Sakura Blocking Solution (10-0032, Sakura) for 2 h, sections were incubated with primary antibodies overnight at 4°C. Endogenous peroxidase activity was blocked using 0.3% H_2_O_2_ for 15 min, followed by incubation with HRP-conjugated secondary antibodies. Signals were developed using DAB substrate and counterstained with hematoxylin. Primary antibodies used were Ly6G (1:50; #127601, BioLegend), ARG1 (1:200; #GTX113131, GeneTex), PLIN2 (1:200; #15294-1-AP, Proteintech), and MSR1 (1:100; #bs-6763R, Bioss Inc). Stained sections were visualized using a NanoZoomer S60 slide scanner (Hamamatsu Photonics).

### Minimal inhibitory concentrations and chemotherapy

MICs were determined by inoculating MAC strains at 2.5 × 10^5^ CFU/mL in mycobacterial medium containing antibiotics in 96-well plates and incubating at 37°C for 5 days. Bacterial growth was assessed by measuring optical density at 600 nm. The MIC for each drug was defined as the lowest concentration preventing any detectable microbial growth. All assays were performed in duplicate to ensure reproducibility. Clarithromycin, ethambutol, and rifampicin were purchased from Tokyo Chemical Industry. For chemotherapy, six mice infected with FKJ-1 or FKJ-8 at 4 weeks p.i. were orally administered a combination of clarithromycin (100 mg/kg), ethambutol (100 mg/kg), and rifampicin (10 mg/kg) in 100 μL of 0.5% methylcellulose solution (Fujifilm) five times per week for 4 weeks. Drug efficacy was quantified as the log10-fold changes in bacterial burden, calculated as log10(CFU_treated_)-log10(CFU_control_). This value corresponds to the log10-transformed ratio between the average CFU counts of treated and control mice. Each experiment was performed in two independent replications to compare the results.

### Statistical analysis

Statistical analyses were performed using ANOVA followed by Tukey–Kramer *post-hoc* test, Mann–Whitney *U* test, and Welch’s *t*-test, as indicated in each figure legend. Adjusted *P* values <0.05 were considered statistically significant. All statistical analyses were conducted using R software (version 4.4). All statistical comparisons are listed in Table S1.

## Data Availability

The data have been deposited with links to BioProject accession number PRJDB37637 in the DDBJ BioProject database.

## SUPPLEMENTAL MATERIAL

Fig. S1 spectrum.03128-25-s0001.tiff First part.

Fig. S1 spectrum.03128-25-s0002.tiff Second part.

Table S1 spectrum.03128-25-s0003.xlsx Tukey-Kramer *post hoc* analysis of the log10-transformed pulmonary CFU counts presented in Fig. 2, 6, 7, and S1.

ASM does not own the copyrights to Supplemental Material that may be linked to, or accessed through, an article. The authors have granted ASM a non-exclusive, world-wide license to publish the Supplemental Material files. Please contact the corresponding author directly for reuse.
