# Supplementary figures and images for: Strain-dependent disease progression and necrotizing granuloma formation in a murine model induced by virulent strains of Mycobacterium avium complex
Source: Microbiol Spectr. 2026 Mar 24;14(5):e03128-25. doi: 10.1128/spectrum.03128-25 (PMC13142028; doi:10.1128/spectrum.03128-25)

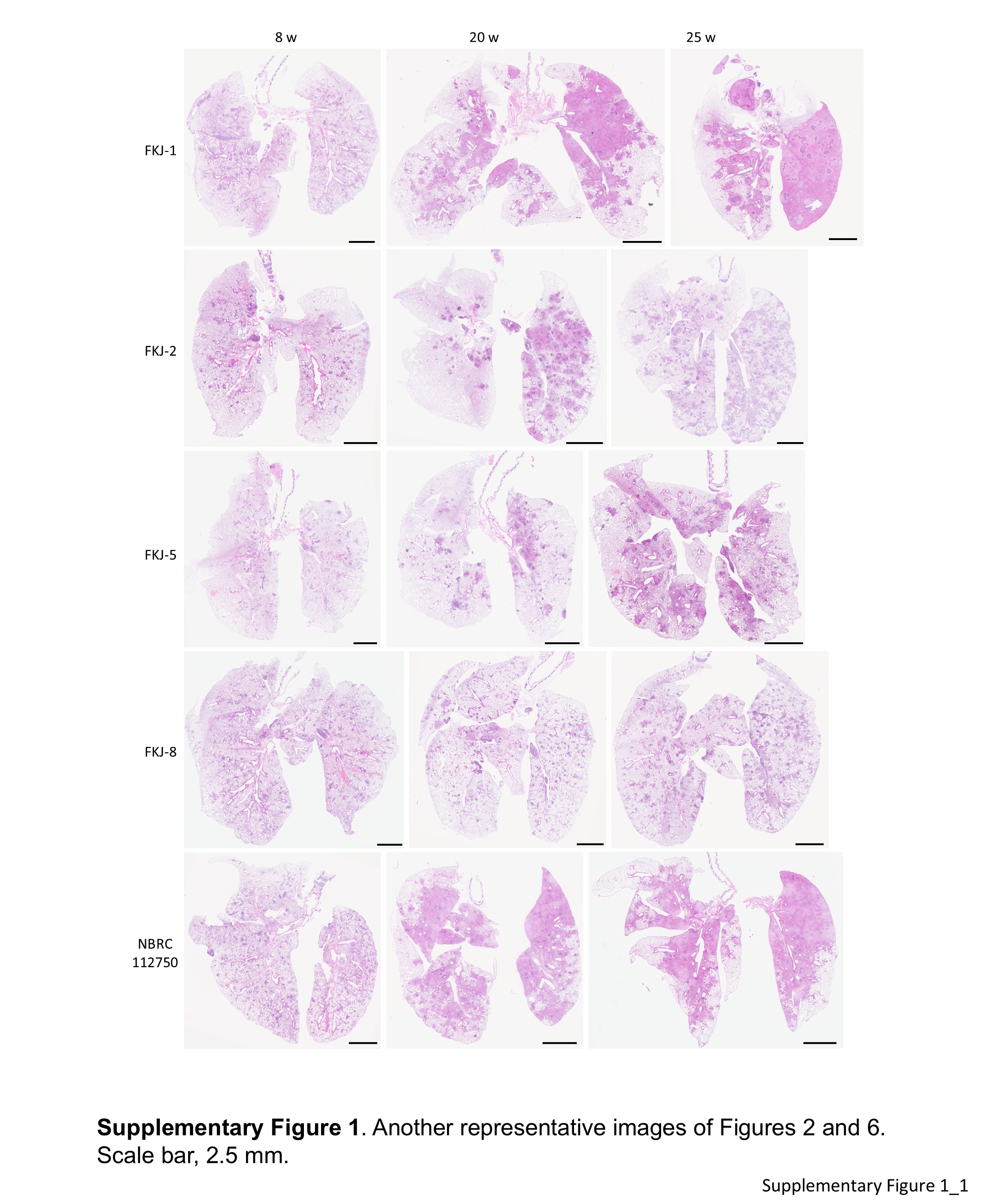

Supplement: Fig. S1 — First part. [file spectrum.03128-25-s0001.tiff]

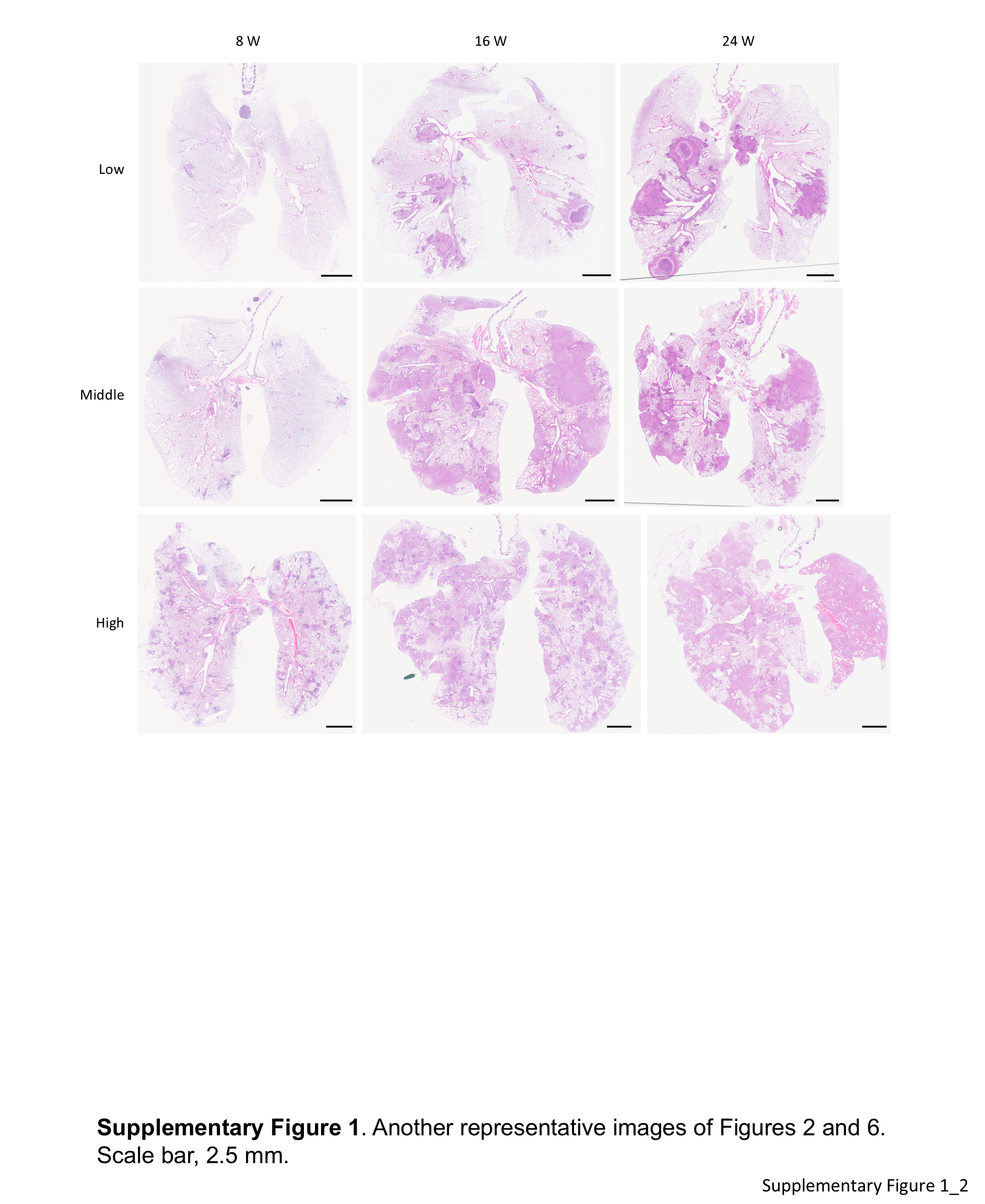

Supplement: Fig. S1 — Second part. [file spectrum.03128-25-s0002.tiff]
